# Stable Isotopes of Carbon and Nitrogen in Female Qamanirjuaq Caribou (*Rangifer tarandus groenlandicus*) Antlers in Relation to Diet and Physiology

**DOI:** 10.1002/ece3.72294

**Published:** 2025-10-07

**Authors:** Matthew Brenning, Fred J. Longstaffe, Joshua H. Miller, Danielle Fraser

**Affiliations:** ^1^ Department of Earth Sciences Carleton University Ottawa Ontario Canada; ^2^ Palaeobiology Canadian Museum of Nature Ottawa Ontario Canada; ^3^ Department of Earth Sciences The University of Western Ontario London Ontario Canada; ^4^ Department of Paleobiology Smithsonian National Museum of Natural History Washington DC USA; ^5^ Department of Geosciences University of Cincinnati Cincinnati Ohio USA; ^6^ Department of Biology Carleton University Ottawa Ontario Canada

**Keywords:** antler, Bayesian stable isotope mixing models, carbon, nitrogen, *Rangifer tarandus groenlandicus*, stable isotope

## Abstract

Populations of Arctic barren‐ground caribou ( *Rangifer tarandus groenlandicus* ) have fluctuated over the past few decades but are currently in decline. To support conservation efforts of caribou, it is integral to develop both historical and modern datasets; these datasets allow us to understand how caribou have adapted to past climatic shifts and may respond to future change. Caribou are the only extant species in which both males and females grow antlers each year, providing unique sex‐specific isotope datasets. Isotopic study of antlers is proving to be a source of annualized data on caribou diet and migration, but uncertainty remains in the magnitude and drivers of isotopic changes across individual antlers. Nitrogen (δ^15^N_Col_) in male antlers from the Qamanirjuaq herd has been shown to increase along the main antler beam aligned with known shifts in seasonal diet and/or the increased energy requirements. We examined 19 female antlers from the same population to compare δ^13^C_Col_ and δ^15^N_Col_ between sexes and among varying reproductive statuses. Female antlers were found to be ~0.4‰ lower in δ^13^C_Col_ at the bases of their antlers compared to males. In both sexes, caribou antler collagen varied in δ^13^C_Col_ among individuals by ~2‰ (−20.0‰ to −18.1‰) and in δ^15^N_col_ by ~5‰ (+2.3‰ to +7.3‰). We conclude that δ^13^C_Col_ differences between sexes are the result of differing diets during the onset of antler formation, corresponding to the different seasons that females and males initiate antler growth. Despite dietary differences, both males and females increased in δ^15^N_Col_ along the antler beam by approximately 1‰–1.5‰. Increases in δ^15^N_Col_ along the antler beam coincide with the increased physiological stress and material demands during antler development. Antler tissue remains a promising resource for studying both short‐ and long‐term changes in male and female caribou ecology.

## Graphical abstract

Isotopic study of antlers is proving to be a source of annualized data on caribou diet and migration, but uncertainty remains in the magnitude and drivers of isotopic changes across individual antlers. We examined 19 female antlers from the Qamanirjuaq population to compare δ^13^C_Col_ and δ^15^N_Col_ between sexes and among varying reproductive statuses. We concluded that while δ^13^C_Col_ in antler tissue correlates with diet, high variation in δ^15^N_Col_ due to physiological stress complicates dietary predictions based solely on δ^13^C_Col_ and δ^15^N_Col_.

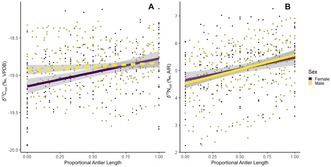

## 1 Introduction

Accelerated climate change in the world's polar regions has had major impacts on local species (Anisimov et al. 2007). This includes barren‐ground caribou ( *Rangifer tarandus groenlandicus* Linnaeus, 1758), a keystone species across much of the Canadian Arctic and a species of high socio‐economic importance to many local Indigenous communities (Wolfe 2004; Mallory and Boyce 2018). Despite strong monitoring efforts by the Canadian federal, provincial, and territorial governments in collaboration with Indigenous and Inuit communities, many populations remain in decline (Barber et al. 2018; Beverly and Qamanirjuaq Caribou Management Board 2014; Festa‐Bianchet et al. 2011). Increases in predation, parasitism, interspecies competition, wildfires, and icing events, as well as climate‐driven shifts in plant assemblages and phenology have led to the degradation and loss of preferred caribou habitats (Mallory and Boyce 2018; Barber et al. 2018; Post and Forchhammer 2008; Theoret et al. 2022). These complex changes to Arctic environments have led to changes in caribou migratory behaviors, changing their departure times, altering grazing routes, and avoiding areas impacted by wildfires or severe ice conditions (rerouting migratory pathways due to freshwater and sea ice breakup) (Mallory and Boyce 2018; Albon et al. 2017; Tews et al. 2007; Le Corre et al. 2017). Populations are impacted by climate on decadal timescales or longer, and the ability of caribou to adapt to climate‐related environmental changes will impact the sustainability of populations (Mallory and Boyce 2018; Miller et al. 2021). Unfortunately, modern datasets alone do not provide the necessary depth for understanding long‐term ecological variability, trends, and cycles under shifting environmental conditions (Miller et al. 2021). Historical records are critical to elucidating the ecological impacts of long‐term environmental changes, including shifts in diet and migration. Antlers and antler tissues have proven to be excellent tools to noninvasively collect both historical and modern data on seasonal diet and migration (Miller et al. 2021, 2013, 2023; Brenning et al. 2024; J. H. Miller 2012; Le Moullec et al. 2019). Caribou are the only extant cervid species in which both sexes develop antlers. Herein, we seek to compare female antler tissue to male and explore how antler tissue may provide unique insight into seasonal diet and migration (Landete‐Castillejos et al. 2019).

Antler growth is triggered by endocrine signals, the hormonal release of testosterone for males and estradiol for females (Blake et al. 1998). During formation, cartilage is laid down on the pedicle bone and mineralized into calcium hydroxyapatite [Ca_10_(PO_4_)_6_(OH)_2_] (Chen et al. 2009). Tissue is laid down in successive layers until the vascularization is stopped, at which point the antler's velvet membrane falls away or is rubbed off, leaving behind dead bone tissue (Chen et al. 2009). While the processes of antler formation are the same for both sexes, the timing and usage of antlers differs. Male caribou begin antler development as early as February, growing large antlers throughout the summer and reaching up to 1.5 m in length, ceasing development in September before the annual rut (Høymork and Reimers 2002). Adult males use their antlers to spar with other males over access to females, and the antlers are cast following the annual rut (breeding period), leaving adult males antlerless in the winter (Høymork and Reimers 2002). In contrast, females begin antler development later in the year. For parturient females, antler growth begins shortly after casting in May or June and continues until October. Females grow comparatively small antlers at a slower rate than males, only reaching up to 0.5 m in length (Espmark 1971). Females retain their antlers throughout the winter, using them to dig through snow to find and defend quality feeding patches (Schaefer and Mahoney 2001; Espmark 1964; Kojola 1989). Females that do not become pregnant, or lose their pregnancy, shed their antlers before the calving period, when parturient females shed their antlers (Espmark 1971, 1964).

For the Qamanirjuaq herd in Northern Canada, antlers are grown by males and females during the annual spring migration as they travel from the boreal forest in northern Saskatchewan to the calving ground on the Arctic tundra in Nunavut (Parker 1972). Migration patterns in the Qamanirjuaq herd differ between sexes. Females and young calves begin their 400 km migration in April, slowly congregating as they travel from their wintering grounds in the boreal forest to Baker Lake (their calving ground) by late August (Beverly and Qamanirjuaq Caribou Management Board 2014; Parker 1972). Male caribou winter in scattered bands, staying south until June, when they begin their migration to the rutting grounds (Parker 1972; D. R. Miller 1976). During migration (e.g., between boreal forest and Arctic tundra), both sexes experience seasonal dietary shifts due to changes in available habitats, as well as seasonal shifts in both the availability and nutritional quality of forage (Beverly and Qamanirjuaq Caribou Management Board 2014; D. R. Miller 1976; Mallory et al. 2020). Typically, the Qamanirjuaq caribou transition from a primarily lichen‐rich diet throughout the winter to one dominated by grasses and grass‐like plants (i.e., sedges and rushes) in the summer (D. R. Miller 1976). In recent years, however, caribou diet has been impacted by Arctic “greening,” a climate‐driven increase in available biomass of vegetation (Post and Forchhammer 2008; Fauchald et al. 2017; Laforge et al. 2023). Increases in vegetation biomass are accompanied by changes to local plant assemblages, including an increase in shrubs with poor nutritional value and, thus, deterioration in the quality of overall caribou pasture (Fauchald et al. 2017). Furthermore, earlier onset of spring greening (vegetation growth) is driving a mismatch between the availability of peak high‐quality food resources and the birth of caribou offspring (Post and Forchhammer 2008; Le Corre et al. 2017; Mallory et al. 2020). The Qamanirjuaq herd's seasonal migration, as with many Arctic herbivores, is cued by local temperature changes, with Arctic warming resulting in earlier departure times. However, when earlier departure occurs, reproductive timing, which is controlled by changes in day length, does not coincide with peak plant growth, resulting in an overall increase in offspring mortality (Beverly and Qamanirjuaq Caribou Management Board 2014; Post and Forchhammer 2008; Le Corre et al. 2017; Mallory et al. 2020). In the face of such complex environmental changes, we need to establish a comprehensive understanding of how ongoing climate changes are likely to impact caribou (Anisimov et al. 2007; Mallory and Boyce 2018; Miller et al. 2021, 2023; Brenning et al. 2024). Only through in‐depth study of historical landscape use will we be able to understand how caribou diet and migration have changed through time. With many caribou populations experiencing instability and even facing extirpation (Festa‐Bianchet et al. 2011; Kaluskar et al. 2020; Tyler 2010), there is a broad need to assess changes in caribou ecology across the last several decades or longer (Miller et al. 2021; Le Moullec et al. 2019; Miller and Simpson 2022).

Antler tissue presents an opportunity to assess yearly and historical shifts in caribou ecology (Miller et al. 2021; Brenning et al. 2024; Stevens and O'Connell 2009). Antlers are unique in that they are developed and shed annually and do not undergo secondary ossification (Chen et al. 2009). Stable isotopes incorporated into developing antler tissues may therefore provide information about seasonal diets and even patterns of landscape use and migration (Miller et al. 2021, 2013; J. H. Miller 2012; Miller and Barry 1992). As antlers can provide ecological assessments of past populations of caribou, it is important to understand the factors impacting isotopic variation within and among antlers, including those linked to sex‐specific differences in ecology and biology.

Herein, we test whether variation in stable carbon and nitrogen isotopes of antler tissues coordinate with known dietary shifts that occur during male and female Qamanirjuaq migrations. Specifically, this companion study aims to expand on observed isotopic patterns found by Brenning et al. (2024) for males in the same population. We expect that the difference in the rate and timing of tissue growth, the variation of diet and foraging space during tissue growth, and the reproductive demands of gestation and lactation create sex‐specific isotopic differences between males and females. We evaluate the patterns of stable isotopes in female Qamanirjuaq caribou antler tissue to understand how they relate to known dietary changes, migration, and overall ecological differences between the sexes within a herd.

Stable isotopes in animal tissues provide valuable information on diet, movement, and physiological condition of both extant and extinct species (Fry 2006; Hobson and Clark 1992; Farquhar et al. 1989; Ehleringer and Rundel 1989; Koch and Phillips 2002). Different isotopes provide proxy data for different ecological characteristics, depending on how those isotopes are incorporated into tissues. For example, carbon and nitrogen isotopes (δ^13^C and δ^15^N) have an array of ecological applications, from reconstructing diet to establishing trophic position and more (Ehleringer and Rundel 1989; Hobson and Wassenaar 1999; Koch 2008; Kelly 2000). The primary source of carbon in animal tissues is diet, with variability dependent on the carbon‐fixing methods of plant photosynthesis (Ehleringer and Rundel 1989; Hobson and Wassenaar 1999; Kelly 2000; Smedley et al. 1991). C_3_ and C_4_ plants have identifiable carbon isotope compositions due to their CO_2_‐fixing enzymes (Farquhar et al. 1989; Kelly 2000; O'Leary 1981). Ribulose 1,5‐bisphosphate in C_3_ plants discriminates against ^13^C isotopes more strongly than phosphoenolpyruvate carboxylase in C_4_ plants, leaving C_4_ plants enriched in δ^13^C relative to C_3_ plants (Farquhar et al. 1989; Kelly 2000; O'Leary 1981). Distinguishing diet can be challenging in Canada's Arctic as the northern boreal forest and Arctic tundra are both dominated by C_3_ plants, which vary in δ^13^C by ~10‰ (−34‰ to −24‰) (Smith and Epstein 1971; Kristensen et al. 2011). Additionally, most food sources of large Arctic herbivorous mammals fall within a δ^13^C range between −28‰ and −24‰ (Brenning et al. 2024; Drucker et al. 2010; Kristensen et al. 2011). There are, however, some discernible patterns within the Arctic flora. Lichens, fungi, and horsetails exhibit higher δ^13^C than woody plants and liverworts found in the same environments (Brenning et al. 2024; Brooks et al. 1997; Drucker et al. 2012; Cardinale et al. 2008; Máguas and Brugnoli 1996). Lichens are dependent on symbiosis with a photobiont whose method of CO_2_ diffusion results in higher δ^13^C than C_3_ vascular plants (Máguas and Brugnoli 1996; Lange et al. 1986). Ectomycorrhizal fungi show higher δ^13^C than their host plants due to isotopic fractionation that occurs during the biochemical process of transferring carbon from host plant to fungus (Högberg et al. 1999; Pate and Arthur 1998). For the Qamanirjuaq herd, dietary changes between February and October—the period of male antler growth—include a shift from higher δ^13^C food sources (i.e., lichen‐dominated) to a mixed diet with lower δ^13^C food sources (i.e., woody and rush plants) (D. R. Miller 1976). Females, however, generally grow their antlers from June to October, during which diets consist almost entirely of grasses, sedges, and rush plants (Parker 1972; D. R. Miller 1976). A deviation to this is females that are not parturient in the prior year, which will initiate antler growth a month earlier (Espmark 1971; Schaefer and Mahoney 2001; Bergerud 1976).

D. R. Miller (1976) collected rumen contents of Qamanirjuaq during three consecutive seasons for 3 years and found no significant intra‐seasonal differences in rumen contents between sexes or among females with varying reproductive conditions. However, he did find significant between‐season differences in rumen content and associated dietary differences. Because lichens typically have higher δ^13^C and lower δ^15^N than the graminoid or grass/grass‐like plants (Brenning et al. 2024; Brooks et al. 1997; Drucker et al. 2012; Milligan 2008), differences in the stable isotope profiles of male and female antlers may differ simply due to the diet during the timing of antler growth. Interpretation of isotopic data, however, can be complex due to a variety of factors, including species‐specific isotopic enrichment during tissue formation, inconsistent isotopic mixing of food sources into tissues, and/or an overrepresentation of protein‐rich foods of δ^13^C in bone collagen due to isotopic routing during tissue synthesis (Gannes et al. 1997; Ambrose and Norr 1993). Brenning et al. (2024) investigated whether male Qamanirjuaq antler tissue synthesis could be used to track spring seasonal dietary change. Specifically, they evaluated the correlation between Miller's observed dietary shifts and the isotopic patterns of collagen δ^13^C_col_ and δ^15^N_col_ in antler. They noted that δ^13^C_col_ varied by ~1‰ within and among individuals and δ^15^N_col_ increased along male antlers by ~1‰–1.5‰, concluding that the changes indicated coordinated seasonal dietary shifts and/or stress of increased energy requirements.

While seasonal diet impacts the isotopic compositions of animal tissues, biological differences between sexes can also result in isotopic variation. Intraspecific competition for food resources can, for example, lead to divergent niches as well as differential growth between males and females, differences in isotope incorporation rates, and differences in reproductive status (Kurle et al. 2014; Kernaléguen et al. 2012; Walter 2014; Rodde et al. 2020; MacAvoy et al. 2012). To establish expectations for how δ^13^C_col_ and δ^15^N_col_ might differ between sexes, it is necessary to understand the possible sources of sex‐based differences. Barren‐ground caribou display sexual segregation and differences in landscape use between males and females, which may lead to differences in average antler isotopic values between sexes (Drucker et al. 2010; Barboza and Bowyer 2001) given that sexual segregation may occur to accommodate sex‐specific dietary and catabolic needs (Barboza and Bowyer 2001; Finstad and Kielland 2011). Reproductive females have higher energy and protein demands; avoiding more aggressive males and selecting areas with high‐quality pastures may help maximize energy intake while minimizing feeding time (Barboza and Bowyer 2001; Weckerly and Ricca 2014). Sex‐specific feeding differences can also arise because female movement and foraging patterns tend to be informed by predator avoidance, whereas males use an optimal foraging strategy (Barboza and Bowyer 2001; Jakimchuk et al. 1987). Sexual segregation decreases prior to mating as males and females congregate (Barboza and Bowyer 2001).

Sex‐based differences in δ^13^C and δ^15^N have been found in soft tissue as a result of sex‐specific hormones that affect growth and metabolic processes (Kurle et al. 2014; Kim et al. 2021). For example, for sexually size dimorphic species, larger individuals require additional N recycling due to increased waste, resulting in large individuals retaining more ^14^N, subsequently lowering tissue δ^15^N relative to smaller‐bodied individuals (Kurle et al. 2014). Contrastingly, size dimorphism does not drive variation in δ^13^C in collagen, as δ^13^C_col_ is largely impacted by amino acids from food sources such as glycine (Kurle et al. 2014; MacAvoy et al. 2012; O'Connell et al. 2001). Antler growth rate and overall biomass, which are both higher for males (Barboza and Parker 2008; Loe et al. 2019), may result in more ^14^N retention, driving a decrease in δ^15^N (Kurle et al. 2014; Fuller et al. 2004; Martínez Del Rio et al. 2009).

Among females, reproductive status can also impact the isotopic compositions of their tissues. Fetal development requires more nutrient resources, and thus nitrogen is retained to accommodate the increased demand from tissue synthesis; retention is attributed to a decrease in urea synthesis, leaving a larger pool of ^14^N within the body, decreasing overall δ^15^N in maternal tissues (Fuller et al. 2004; Kalhan et al. 1998). As antler development begins soon after calving in June and continues as female caribou lactate, δ^15^N of antler tissues may be depleted among reproductive females (Barboza and Parker 2008; Loe et al. 2019; Borrell et al. 2016). In contrast to the fluctuations in δ^15^N caused by pregnancy, δ^13^C is not impacted by fetal growth, as carbon sources are directly used for protein production (Fuller et al. 2004; Borrell et al. 2016; DeNiro and Epstein 1978). We predict that both sex‐based differences in the timing of tissue development will produce distinct antler isotope compositions between males and females. We further predict differences in antler isotope compositions between parturient vs. nonparturient females. Additionally, we predict that lichen‐rich diets of males that coincide with early portions of their antler development will result in higher δ^13^C values in the proximal portions of male antlers compared to those of female antlers. Finally, we predict that female antlers will have lower δ^15^N values, resulting from the high and concurrent nutrient demands of antler production and lactation.

## 2 Methods and Materials

### 2.1 Sampled Caribou

Antlers used in this study were selected from specimens collected by the Canadian Wildlife Services (CWS) during the mid to late 1960s and curated at the Canadian Museum of Nature (CMN). The CWS collection contains 999 individual caribou skeletons, 943 of which belong to the Qamanirjuaq herd (Parker 1972; D. R. Miller 1976; F. L. Miller 1974). The collections were part of a CWS study to better understand the population dynamics of the Qamanirjuaq caribou after years of population decline in the 1940s and 1950s (Parker 1972; D. R. Miller 1976; F. L. Miller 1974). During the almost three‐year survey, the CWS studied herd size, movement, and diet, as well as individual growth, reproduction, and energy reserves (Parker 1972; D. R. Miller 1976; F. L. Miller 1974; Dauphine 1976). Caribou were culled at 12 different periods throughout the study. Sex, age, weight, body length, and all tooth sizes and wear stages were recorded for culled individuals (Parker 1972; D. R. Miller 1976; F. L. Miller 1974; Dauphine 1976). Rumen contents were recorded for 340 of these individuals (D. R. Miller 1976). For females, reproductive status was also recorded (categorized as pregnant, lactating, and dry [nonparturient]). Skulls (including antlers) and teeth were collected. Prior to being accessioned in the museum, tissues were removed from the skulls using a stiff‐bristle brush after soaking in hot water for several days (F. L. Miller 1974). For this study, the antlers of 19 females were selected for similarity in age (> 46 months), body size (~90 kg), culling period (September 1967), and differences in reproductive status (lactating and dry; Table S1). Male antlers used for comparison were sampled by Brenning et al. (2024); they employed a similar methodology for sample selection, studying 13 males of similar age (~50 months), body size (~125 kg), and culling period (September 1967).

### 2.2 Plant Samples

The plant, lichen, and fungi stable isotope dataset used in this study originates from two studies: Brenning et al. (2024) and Hobbie et al. (2017). The combined dataset contains 90 different specimens of 38 species (from 11 genera). Brenning et al. (2024) sampled 78 herbarium specimens (32 different species and 8 different genera) curated in the National Herbarium of Canada (NHC) at the CMN. Plant and lichen species were selected based on proximity to the Qamanirjuaq habitat range and relative age from 1965 to 1968. Additionally, all sampled plant species had been observed in the rumen contents and similarly classified into functional groups (liverwort, lichen, conifer, woody, and grass‐like plants) by the CWS (F. L. Miller 1974). Sufficient fungi specimens were absent from the NHC collection; thus, literary data supplemented the missing functional group. Hobbie et al. (2017) collected 12 fungi samples during an isotopic study on plants and fungi in Arctic ground squirrel diet (Table S3).

### 2.3 Stable Isotope Analysis

All 19 antlers selected for analysis were prepared for collagen carbon (δ^13^C_col_) and nitrogen (δ^15^N_col_) isotopic analysis. The surface of the antler was cleaned by removing at least 3 μm of tissue using an 8220 Micro 12Vmax High Performance Cordless Dremel tool with a 3/32‐in. Diamond Wheel Taper Point Rotary Bit. The cleaned antler surface was then drilled, collecting 4 mg of powdered antler tissue every 5 cm along the beam of each antler for a total of 159 samples taken from the 19 antlers. Collagen extraction followed the same method used by Brenning et al. (2024) on male caribou antlers. For each sample, 2–3 mg of dried antler powder was weighed out and placed into plastic microcentrifuge tubes with 1.5 mL of 0.1 M HCl solution (initial procedure suggested 1 M HCl but was reduced to avoid the loss of nitrogen). Tubes were left open but covered with aluminum foil caps and placed into a refrigerator for 30 min to decalcify. Published research procedures suggested a 2‐day decalcification period, but this process caused the complete degradation of antler powder material and loss of nitrogen (Trayler et al. 2023). The resulting solution was aspirated and then rinsed using 1 mL of deionized water, which was subsequently agitated, centrifuged, and aspirated again. The wash process was done five times for each sample before samples were placed in a freeze dryer overnight to remove remaining water. Usual collagen preparation procedures suggest defatting; however, this step was omitted as antler do not contain enough fat to require this procedure (Chen et al. 2009).

Element composition and stable isotopes of carbon and nitrogen were measured at the Laboratory of Stable Isotope Science (LSIS), at The University of Western Ontario. All isotopic results are reported using the typical delta (δ) notation in parts per thousand (‰) relative to VPDB and AIR. All analytical errors in the present study are reported to one standard deviation (1*σ*).

About 0.3–0.5 mg of antler collagen from each sample and suitable standards were weighed into tin capsules. Capsules were crimped and loaded into an autosampler attached to a Costech ECS 4010 elemental analyzer (EA) interfaced with a Thermo Scientific Delta^PLUS^ XL isotope ratio mass spectrometer (IRMS). Using helium as a carrier gas, samples and standards were combusted and released into the IRMS in continuous‐flow (CF) mode. Standards were analyzed at the beginning and end of each analytical session (6 in total) and after every five samples; all sessions were free of instrumental drift. Stable carbon and nitrogen isotope compositions, the amount (wt.%) of each element, and the C/N ratio for each sample and standard were collected within the same analytical session.

Isotopic ratios were calibrated to VPDB and AIR using USGS40 (l‐glutamic acid; *n* = 17; 1*σ* δ^13^C = 0.03‰, accepted δ^13^C = −26.39‰; 1*σ* δ^15^N = 0.09‰, accepted δ^15^N = −4.52‰) and USGS41a (l‐glutamic acid; *n* = 17; 1*σ* δ^13^C = 0.50‰, accepted δ^13^C = +36.42‰; 1*σ* δ^15^N = 0.23‰, accepted δ^15^N = +47.55‰). The accuracy of the calibration curve was tested using the LSIS internal standard (keratin, MP Biomedicals Inc., Cat No. 90211, Lot No. 9966H) (measured δ^13^C = −24.08‰ ± 0.06‰, *n* = 36; measured δ^15^N = +6.44‰ ± 0.08‰; *n* = 36), which compared well with its accepted values (δ^13^C = −24.05‰; δ^15^N = +6.40‰; *n* = 1999). The calibration curve accuracy was also further tested using Szpak SRM‐14 (measured δ^13^C = −13.72‰ ± 0.05‰, *n* = 12; measured δ^15^N = +21.53‰ ± 0.20‰; *n* = 13), which compared well with its accepted values (δ^13^C = −13.68‰ and δ^15^N = +21.62‰). Duplicates of samples differed by an average of 0.06‰ ± 0.07‰ for δ^13^C (*n* = 14) and 0.23‰ ± 0.52‰ for δ^15^N (*n* = 14). The content of collagen carbon in samples averaged 42.69 ± 4.22 wt.% and 14.85 ± 1.46 wt.%, respectively (*n* = 174). The average C/N ratio of samples was 3.35 ± 0.08 and the average difference between C/N ratios of duplicates was 0.03 ± 0.04 (*n* = 14) (Table S2). All C/N ratios fell within the accepted range of fresh/well‐preserved collagen (i.e., 2.9–3.6) (DeNiro 1985).

### 2.4 Statistical Analysis

A Shapiro–Wilk's test indicated that antler δ^13^C_col_ and δ^15^N_col_ values for both females and males had a nonnormal distribution. Thus, we compared stable isotope compositions between sexes and among reproductive statuses for females using the nonparametric Wilcoxon signed‐rank test. Comparisons were made between the average antler δ^13^C_col_ and δ^15^N_col_ values throughout the whole antler as well as the averages from both the base and tip of the antler.

Bayesian stable isotope mixing models (BSIMMs) were used to determine caribou diets via antler δ^13^C_col_ and δ^15^N_col_ compositions. To establish an effective BSIMM, strong knowledge of the consumer's diet is required (Moore and Semmens 2008; Stock et al. 2020, 2018). BSIMMs can only evaluate food sources that are included in the model; thus, any omitted food source will negatively impact the models' utility (Moore and Semmens 2008; Stock et al. 2020, 2018). Priors can be added to the model, providing opportunities to weight candidate food sources based on known dietary composition. For this analysis, the dietary mix used was the same as in Brenning et al. (2024); food sources were divided into five categories: fungi, horsetail (grass‐like plants; grasses, sedges, and rushes), liverwort, lichen, and woody plants. The dietary mix was based on rumen contents and observed feeding behaviors taken prior to the original culling of individuals (D. R. Miller 1976). All food sources indicated by CWS were included in the dietary mix. The use of five dietary categories in the mixing model was used to minimize isotopic overlap in competing food sources, which tend to be isotopically (δ^13^C and δ^15^N) similar in Arctic forage. Brenning et al. (2024) found that dividing the caribou food sources into five functional categories produced the highest degree of isotopic separation among candidate dietary sources.

Mixing models were run in RStudio using MIXSIAR (version 2022.12.0 + 353, R Core Team 2020; Stock et al. 2020), four models were employed, each with a different set of priors and covariates. Of the four models, two were set with informative priors: one with the covariate of antler length (isotope sample position along the beam of the antler) and the other with no random or fixed effects. Informative priors were based on the percentages of each functional group found in the rumen content of culled caribou (informative priors were set as the following: fungi 0.15, horsetail 0.20, lichen 0.09, liverwort 0.13, and woody plants 0.42) (D. R. Miller 1976). The remaining two models were both set with uninformative priors; similarly, one model used antler length as a covariate while the other had no random or fixed effects. Mixing model results were then compared using Leave‐One‐Out Information Criterion (LOOIC) to help determine which combination of candidate dietary sources best fit the antler isotope data. To determine if caribou diet varied in accordance with sampling position along the antler beam, we set isotope values along the antler lengths as a continuous variable within the mixing model. Following Brenning et al. (2024), all mixing models were set with the following parameters: number of chains = 3, chain length = 100,000, burn = 50,000, thin = 50.

## 3 Results

### 3.1 Isotopic Composition of Sexes

**FIGURE 1:**
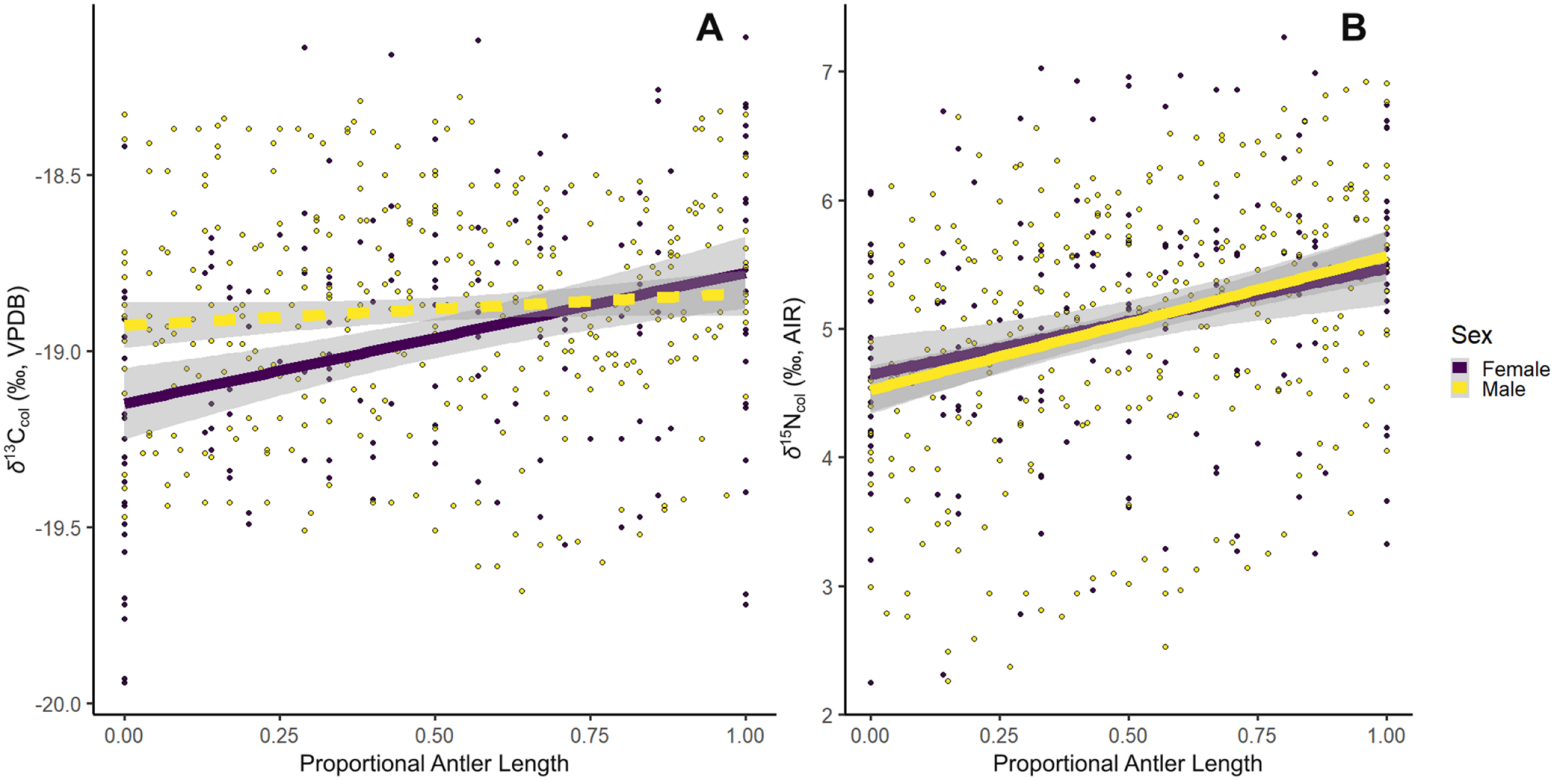
Linear regression analysis for Qamanirjuaq male and female antler carbon and nitrogen isotopic variability across the proportional length of the beam; position 0.00 represents the base of the antler and 1.00 represents the tip. (A) δ^13^C_col_ and (B) δ^15^N_col_ linear regression with 95% confidence intervals of isotopic variation along the antler beam for all individuals based on sex; females (purple) and males (yellow). Solid and dashed lines indicate statistically significant and nonsignificant relationships, respectively (Table S4).

Antler collagen for female Qamanirjuaq caribou varied among individuals by ~2‰ for δ^13^C_col_ and ~5‰ for δ^15^N_col_ (−20.0‰ to −18.1‰ and +2.3‰ to +7.3‰, respectively). Along individual antler beams, δ^13^C_col_ varied by 1‰ and δ^15^N_col_ by 1.4‰. Antlers from males showed a similar range within and among individuals, with δ^13^C_col_ varying by < 0.6‰ within an antler beam and ~1.5‰ among individuals and δ^15^N_col_ varying by ~2‰ within an antler beam and ~4.5‰ among individuals. In both sexes, δ^13^C_col_ and δ^15^N_col_ varied considerably among individuals. δ^13^C_col_ did not show consistent increases along the antler beam (i.e., males had nonsignificant results), while δ^15^N_col_ did significantly increase towards the antler tip for both sexes (Figure 1). The median δ^13^C_col_ value of antlers from females was significantly lower than for males (i.e., −18.96‰ ± 0.38‰ and −18.88‰ ± 0.31‰, respectively; Wilcoxon signed‐rank test, *p* = 0.021; Table 1, Figure 1A); however, this difference may not be biologically (or, at least, interpretationally) meaningful. δ^15^N_col_ was not significantly different between female and male antlers (+5.05‰ ± 1.05‰ and +5.05‰ ± 0.96‰, respectively; Wilcoxon signed‐rank test, *p* = 0.904; Table 1, Figure 1B). Neither δ^13^C_col_ nor δ^15^N_col_ showed significant variation between antlers of females of different reproductive status (Table 1).

**TABLE 1:** Statistical comparisons of both δ^13^C_col_ and δ^15^N_col_ (‰) between sexes and among reproductive statuses using the Wilcoxon signed‐rank test. Comparison includes average antler isotope compositions for both sexes, average antler tip isotopes for both sexes, and average antler base isotopes for both sexes. An additional statistical comparison was made between average antler base and tip isotope values regardless of sex.

Wilcoxon signed‐ranked test										
Isotope	Sex	No of samples	Mean (‰)	SE	Sex	No of samples	Mean (‰)	SE	Statistics	*p*
δ^13^C_col_	Female	159	−18.96	0.03	Male	344	−18.88	0.02	23,861	0.0214^a^
	Female—Tip	23	−18.83	0.09	Male—Tip	13	−18.73	0.06	132	0.575
	Female—Base	23	−19.31	0.08	Male—Base	15	−18.98	0.09	90.5	0.0149^a^
δ^15^N_col_	Female	159	+5.06	0.08	Male	344	+5.04	0.05	27,168	0.906
	Female—Tip	23	+5.35	0.19	Male—Tip	13	+5.57	0.23	132	0.587
	Female—Base	23	+4.50	0.19	Male—Base	15	+4.47	0.19	176	0.929

	Male and female				Male and female					
δ^13^C_col_	Base	38	−19.18	0.06	Tip	37	−18.80	0.06	318	< 0.0001^a^
δ^15^N_col_	Base	38	+4.49	0.14	Tip	37	+5.45	0.14	305	< 0.0001^a^

	Female: dry and lactating				Female: dry and lactating					
δ^13^C_col_	Dry	91	−18.96	0.04	Lactating	68	−18.96	0.04	3037	0.844
	Dry—Tip	13	−18.81	0.11	Lactating—Tip	10	−18.86	0.15	70.5	0.756
	Dry—Base	14	−19.26	0.12	Lactating—Base	9	−19.39	0.10	73	0.557
δ^15^N_col_	Dry	91	+5.19	0.11	Lactating	68	+4.89	0.12	3588	0.086
	Dry—Tip	13	+5.54	0.27	Lactating—Tip	10	+5.09	0.28	95	0.067
	Dry—Base	14	+4.53	0.23	Lactating—Base	9	+4.45	0.36	61	0.925

^a^ Statistically significant results.

### 3.2 Bayesian Stable Isotope Mixing Models (BSIMMs)

Combining all antlers from females, the BSIMMs returned a mixed diet for females, with little to no change in posterior estimates among the different food sources across antler beams (Figure 4A). All estimated dietary proportions for the five categories had high uncertainty (i.e., large 95% credible intervals) and generally high overlap (Table 2; Figure 4, Figure S1). Diet is generally reconstructed as mixed, with no strong differences observed among antlers. The only notable changes going from antler bases to tips were slight (but nonsignificant) decreases in woody plants and an increase in lichens. In general, model results indicate liverworts in caribou diet. Both uninformative (Figure 4, top row) and informative priors (Figure 4, bottom row) produced similar results, with greater uncertainty in the estimated proportion of diet in the model with informative priors (Figure 4F–H). Model results using informative priors also estimate lower amounts of horsetail and fungi in caribou diets (Table 2; Figure S1). Mixing models were also compared using LOOIC. The best fit of these models used antler length as a continuous covariate and uninformative priors (Table 3).

**TABLE 2:** Bayesian stable isotope mixing model mean probabilities with 95% credible intervals.

Mean—95% credible interval					
Model	Fungi	Horsetail	Lichen	Liverwort	Woody
Uninformative no effects	0.222–0.285	0.180–0.244	0.264–0.327	0.028–0.051	0.307–0.372
Uninformative antler length continuous covariate	0.224–0.290	0.181–0.245	0.264–0.326	0.027–0.054	0.304–0.370
Informative no effects	0.177–0.268	0.075–0.219	0.225–0.312	0.293–0.549	0.229–0.351
Informative antler length continuous covariate	0.074–0.262	0.154–0.311	0.314–0.497	0.200–0.630	0.259–0.360

**TABLE 3:** Bayesian stable isotope mixing models compared using Leave‐One‐Out Information Criterion (LOOIC), the difference between each model and the model with the lowest LOOIC, and calculated Akaike weights (estimated probability that the model will make the best prediction on new data).

Models		LOOIC	SE	Difference LOOIC	SE difference LOOIC	Akaike weight
Priors	Antler length					
Uninformative	Continuous covariate	4.4	9.3	0.0	NA	0.776
Uninformative	No effect	6.9	9.8	2.5	3.3	0.222
Informative	Continuous covariate	17.6	8.9	13.2	1.0	0.001
Informative	No effect	56.7	7.3	52.3	3.8	0.000

## 4 Discussion

In spite of strong sex‐based distinctions in caribou migratory ecology, the timing of antler development, and reproductive demands (Parker 1972; D. R. Miller 1976), we found only modest differences in their antler isotopic values. Female antlers showed only slightly lower δ^13^C_col_ values relative to males. We also found strong similarity in δ^15^N_col_ among caribou of differing reproductive statuses (i.e., male, female—dry, or female—lactating) (Table 1), with sexes exhibiting a similar increase in δ^15^N_col_ from antler bases to tips. In alignment with our predictions, males showed higher δ^13^C_col_ (by ~0.4‰) than females at the antler base (Table 1; Figure 1A). The difference in δ^13^C_col_ between sexes decreased along the antler beam, with the antler tip showing no significant difference between sexes. Offsets in δ^13^C_col_ at antler bases but not for antler tips are consistent with sex‐based differences in the onset of antler growth (February for males and May for females) correlating with different seasonal diets as observed by D. R. Miller (1976) in rumen content. Additionally, Qamanirjuaq rumen content exhibits enhanced dietary similarities during the finalization of annual antler growth (September for males, October for females). It is likely that the base of male antlers reflects their winter diet rich in lichens (relatively high δ^13^C), while the base of female antlers reflects a mixed summer diet of lower δ^13^C woody and grass‐like plants. Towards the end of antler growth, when the tip is forming, both sexes are consuming a mixed diet of lichen, fungi, grass‐like, and woody plants (D. R. Miller 1976) (Table 1; Figure 1A).

All male and female Qamanirjuaq caribou (except one: CMN391298) increased in δ^15^N_col_ along the antler beam from the base to the tip (by ~1‰–1.5‰) (Figure 1B). However, among individuals sampled, average antler δ^15^N_col_ exhibited a large range (~2.25‰–6.07‰). Variation in δ^15^N_col_ among individuals may result from how different Arctic food sources (i.e., lichen, plants, and fungi) uptake the limited nutrient nitrogen in northern Canada (Drucker et al. 2010; Kristensen et al. 2011). Arctic food sources display a range of δ^15^N isotope values due to the soil depth at which they source nitrogen; at different soil depths, the rates of denitrification, mineralization, and leaching change (Nadelhoffer et al. 1996; Cabello et al. 2009). Additionally, how Arctic food sources accumulate their nitrogen (i.e., from the soil, precipitation, or runoff) and how nitrogen is fixed can result in wide‐ranging δ^15^N in Arctic food sources (Nadelhoffer et al. 1996; Cabello et al. 2009). Lichen, the dominant food source for caribou during the winter months, tends to be more depleted of ^15^N compared to grasses, sedges, and rushes (D. R. Miller 1976; Kristensen et al. 2011; Schulze et al. 1994; Denryter et al. 2017; Aerts et al. 2009; Gustine et al. 2014). Brenning et al. (2024) concluded that winter to summer changes in diet from lower to higher δ^15^N foods (i.e., lichen to grasses, sedges, rushes, and woody plants) correlated with observed changes in rumen content and may have driven the observed δ^15^N_col_ increases in male Qamanirjuaq antlers (D. R. Miller 1976). Females, however, begin antler growth in the summer, when diets have already shifted away from lichens (Parker 1972; D. R. Miller 1976), suggesting that observed δ^15^N_col_ patterns in antler tissue would be different between sexes if diet were the primary influencer. Furthermore, the similar δ^15^N_col_ patterns in male and female antlers suggest that isotopic composition does not reflect sex‐specific catabolic differences that would result from the different timing of antler growth onset (Finstad and Kielland 2011).

**FIGURE 2:**
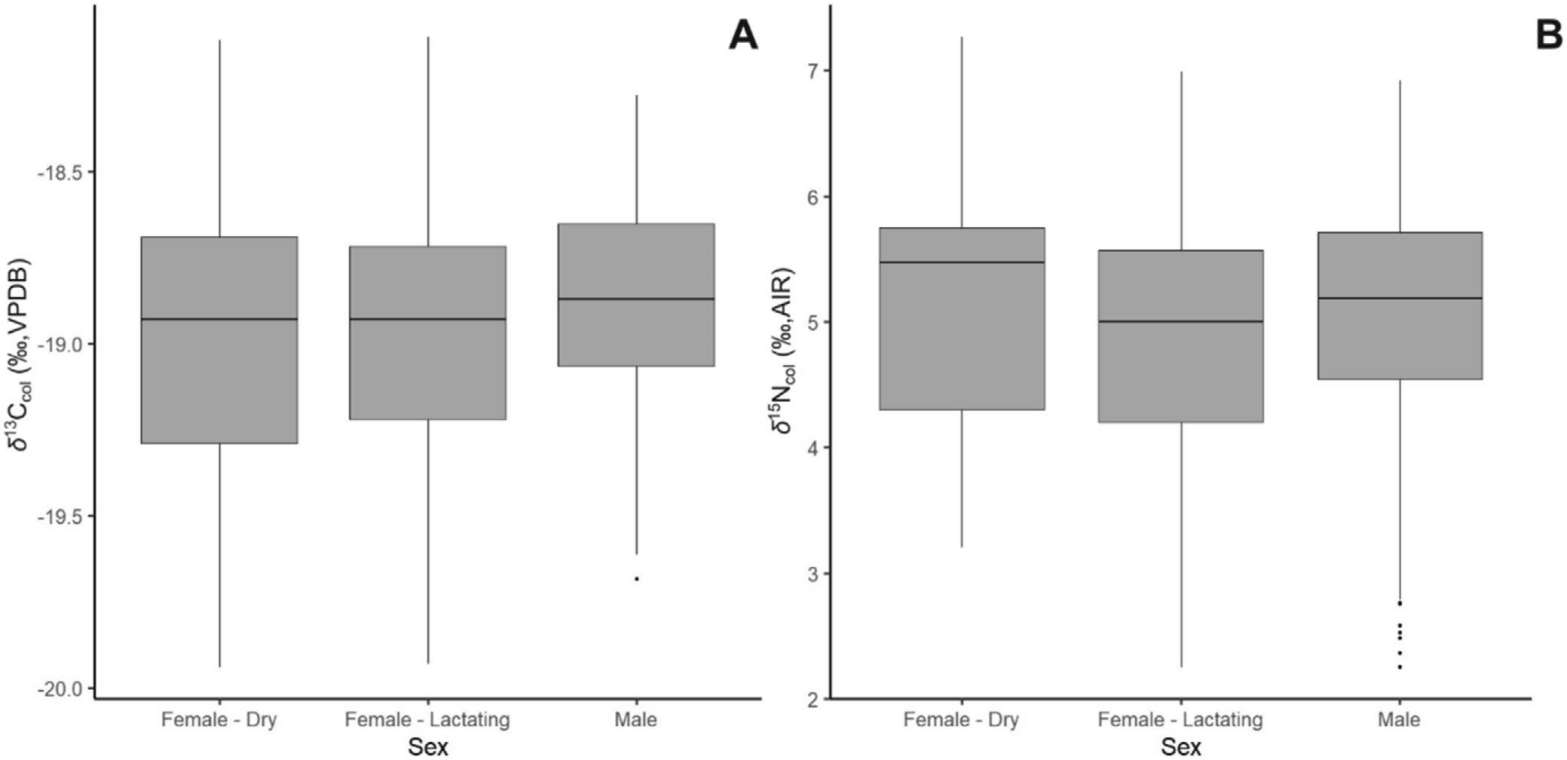
(A) δ^13^C_col_ and (B) δ^15^N_col_ of antler compared to their reproductive status: female—dry, female—lactating, and males.

**FIGURE 3:**
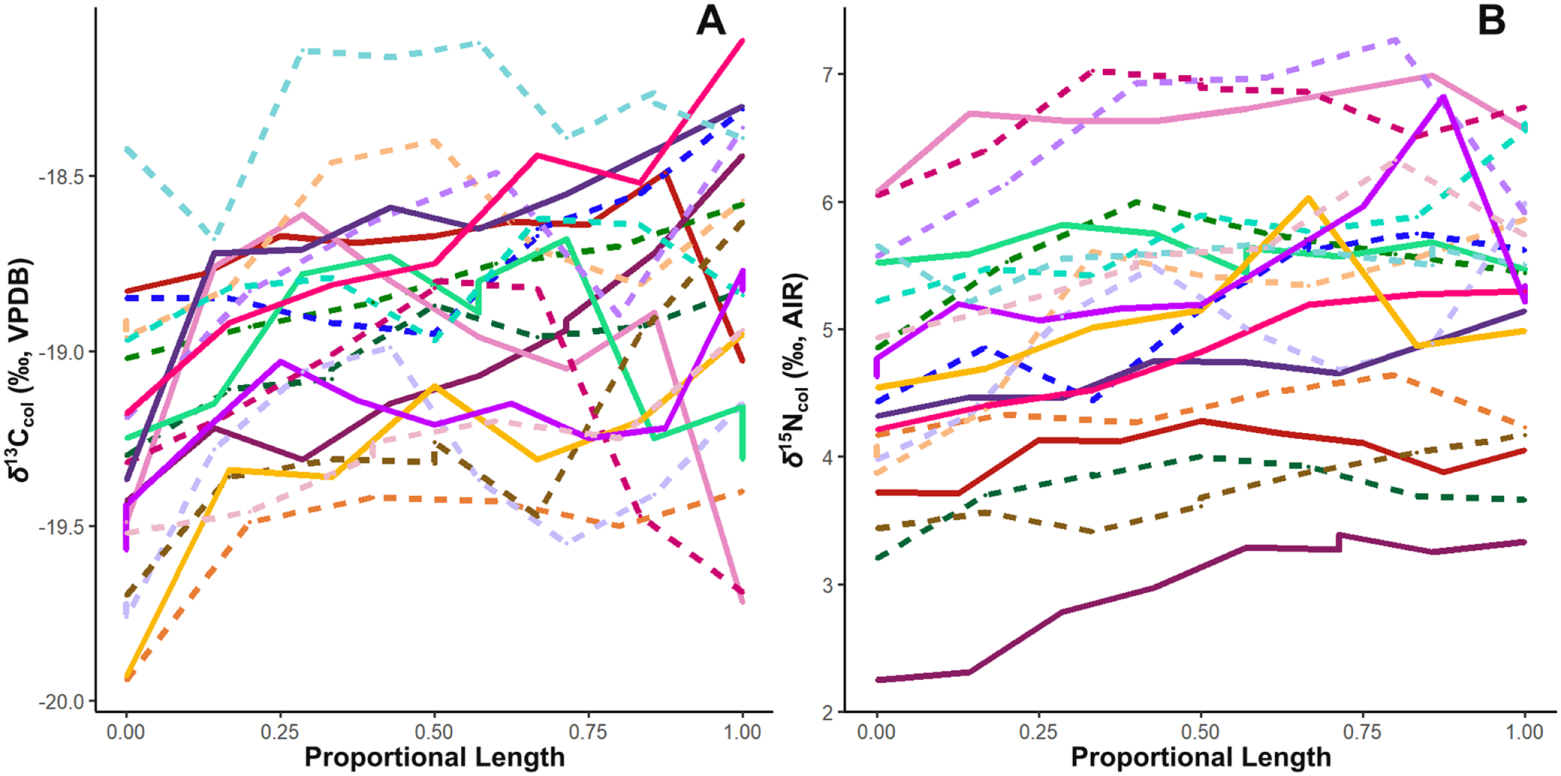
Individual female carbon and nitrogen isotopic variability across the proportional length of an antler beam; position 0.00 represents the base of the antler and 1.00 represents the tip. (A) δ^13^C_col_ and (B) δ^15^N_col_ isotope trends each individual female—dashed for lactating females, solid for dry females.

Antler growth is characterized by rapid tissue formation and requires large amounts of energy, calcium, and phosphorus (Baksi and Newbrey 1989; Moen and Pastor 1998). To compensate for the increased demand and high metabolic stress, caribou will reabsorb minerals from their ribs and other skeletal bones, with lost skeletal density only being repaired after antler development (Baksi and Newbrey 1989; Moen and Pastor 1998). Recycling of nitrogen can also occur in the rumen during periods of increased demand; urea, used in microbial digestion, can be recycled for additional nitrogen absorption, thus ^15^N that would normally be removed as waste is retained and potentially used for collagen synthesis in antler growth (Kelly 2000; Ambrose 1991; Sealy et al. 1987; Finstad and Kielland 2011). The combination of increased physiological stresses and material demands during antler tissue development could be a driving factor in ^15^N enrichment along antler beams for both sexes (Hobson and Clark 1992; Kelly 2000; Ambrose 1991). Reproductive differences were expected to drive δ^15^N decreases within female antlers, as lactation continues for ~6 months following calving and has been shown to decrease in δ^15^N_col_ in maternal tissues (Barboza and Parker 2008; Loe et al. 2019; Borrell et al. 2016; Dauphine 1976). However, no significant differences were found between dry and lactating female antlers (Table 1; Figures 2 and 3).

**FIGURE 4:**
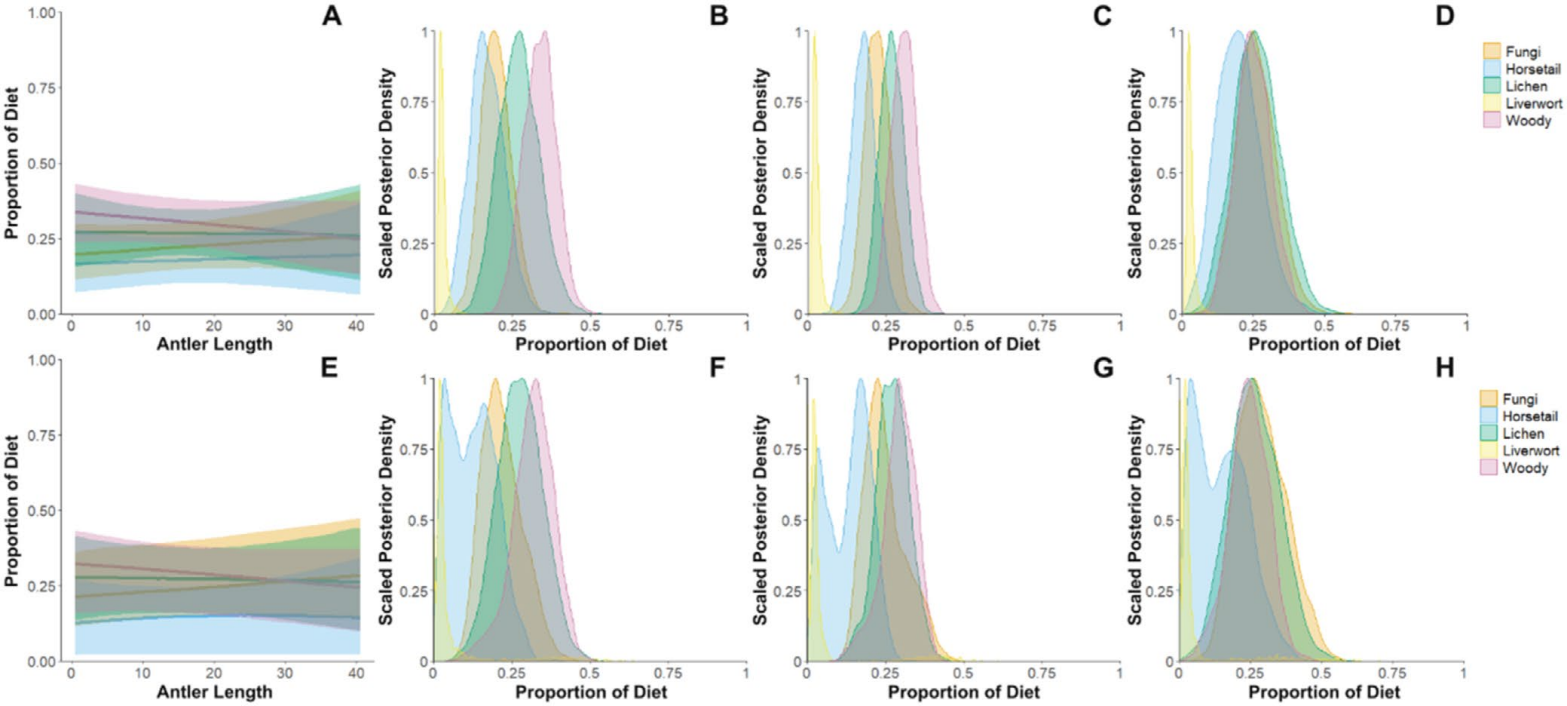
Posterior distributions for female caribou diet proportions as a function of length from two mixing models (A) Uninformative priors: Estimating a heavily mixed diet with a high degree of uncertainty and (E) Informative priors: Estimating a horsetail, lichen, and woody plant mixed diet with a high degree of uncertainty. (B–D) The probability distributions of each food source at different segments of the antler using the mixing model with uninformative priors. (B) Base of antler: A mixed diet of slightly more woody plants and lichen with no liverwort. (C) Middle of antler: A mixed diet of slightly more woody plants and lichen with no liverwort. (D) Tip of antler: An equally mixed diet of fungi, horsetail, lichen, and woody plants. (F–H) The probability distributions of each food source at different segments of the antler using the mixing model with informative priors. (F) Base of antler: a mixed diet of liverwort and woody plants. (G) Middle of antler: a mixed diet of liverwort and woody plants. (H) Tip of antler: a mixed diet of liverwort, woody plants, and lichen.

Bayesian Stable Isotope Mixing Models (BSIMMs) of antler δ^13^C_col_ and δ^15^N_col_ values were expected to reconstruct seasonal dietary shifts in males and more muted dietary shifts for females. However, our findings were inconclusive. Brenning et al. (2024) similarly employed BSIMMs for male caribou antlers, inferring slight (but nonstatistically significant) dietary changes from lower δ^15^N foods (i.e., lichen) to higher δ^15^N foods (i.e., grass‐like plants). Herein, BSIMMs returned largely mixed diets with slight, but nonstatistically significant, decreases in dietary woody plants and increases in lichen during the formation of antler tips (Figure 4). Such reconstructed dietary shifts are consistent with females incorporating more lichens into their diets during the fall, which has been recorded in stomach contents (D. R. Miller 1976). However, as reported by Brenning et al. (2024), high uncertainty in model outputs (Figure 4; Table 2), likely driven by the isotopic overlap of candidate food sources, limits their diagnostic capabilities (Brenning et al. 2024).

There was individual variation among all Qamanirjuaq caribou, regardless of sex and reproductive status. For δ^15^N_col_ and δ^13^C_col_, antlers varied by ~5‰ (~+3‰ to +7‰) (Figure 1B) and ~−2‰ (~−20‰ to −18‰), respectively. In a generalist population (i.e., the Qamanirjuaq herd), the assumption is that all individuals sample equally across the diet distribution; however, individual niche spaces can significantly vary stable isotope compositions among individuals' tissue (Matich et al. 2021). Such variation is, however, consistent with differences in foraging behavior (Rioux et al. 2022). Individualized foraging behaviors could have a significant impact on the δ^15^N_Col_ and δ^13^C_col_ in antlers (Jakimchuk et al. 1987; Rioux et al. 2022; Van Der Wal et al. 2000). Caribou exhibit a high degree of behavioral plasticity, shifting foraging strategies based on food resource availability, inter and intraspecific competition, and predator avoidance (Rioux et al. 2022; Parker et al. 2009). Individualized foraging strategies become more prominent when population density is high (i.e., when caribou band together during migration) (Kaluskar et al. 2020; Rioux et al. 2022; Parker et al. 2009; Le Corre et al. 2020; Pérez‐Barbería et al. 2008). Flexibility in caribou feeding strategies allows them to maximize their fitness, either by obtaining forage from less nutritious food sources to avoid predation or by traveling longer distances to seek highly nutritious, energy‐rich resources like fungi and deciduous shrubs (Rioux et al. 2022; Parker et al. 2009; Le Corre et al. 2020). Individuals may also weigh the cost of competition over the length of migration, migrating further and consuming less nutritious food sources but avoiding competition (Parker et al. 2009; Alonso et al. 1994; Flint et al. 2008). Such foraging flexibility affects a caribou's individual performance, which shifts their δ^15^N_Col_ and δ^13^C_col_ values in developing tissues (Rioux et al. 2022). For the Qamanirjuaq, the wide range of average δ^15^N_col_ and δ^13^C_col_ antlers values may therefore be a result of performance differences, resulting from individual foraging and migratory strategies.

Antler tissue is a valuable tool for noninvasively collecting historical and modern data on seasonal diet and migration. Long‐term dietary shifts may be detectable through antler δ^13^C_col_ values, which could be applied to historical datasets, as climate change over decadal or longer timescales has influenced the availability of food sources (e.g., shifts from grass‐like plants to lichen) (Rivals and Solounias 2007; Zimov et al. 1995). However, predicting diet distributions based on δ^13^C_col_ and δ^15^N_col_ alone remains challenging, as increased physiological stress can mute the expected isotopic shifts associated with dietary changes. Additionally, significant individual isotopic variation suggests that a large dataset would be necessary to create robust assessments of long‐term caribou population trends (Gannes et al. 1997; Martínez Del Rio et al. 2009; Newsome et al. 2012). Despite these challenges, antlers remain an accessible resource that may provide valuable insights into the historical ecological impacts on caribou sustainability and their ability to adapt to future change.

## 5 Conclusions

Using historical collections of caribou from the Qamanirjuaq herd, we compared the δ^13^C_col_ and δ^15^N_col_ of antlers between sexes and found that antler collagen from female Qamanirjuaq caribou was not isotopically distinct from males, except at antler bases where males had ~0.4‰ higher δ^13^C_Col_. This offset correlated with known rumen content changes and is likely due to seasonally influenced dietary differences between males and females when antler growth is initiated. Despite individual variability (~2‰ for δ^13^C_col_ and ~5‰ for δ^15^N_col_), there were no isotopic differences between lactating and nonlactating females. There was a consistent increase in δ^15^N_col_ along antlers, likely a result of increased physiological demands and nitrogen recycling during antler development. Though Bayesian Stable Isotope Mixing Models showed a high degree of uncertainty due to overlapping food source isotope compositions, the significant individual variation in antler isotope compositions highlights the behavioral plasticity of caribou and their use of diverse foraging strategies. These findings illustrate the value of antler tissue as a nondestructive, historical source of caribou ecological information, with strong potential for detecting both short‐ and long‐term ecological shifts.

As climate change accelerates in northern ecosystems, continued isotopic monitoring of antler tissue offers a potentially powerful tool for tracking changes in forage availability, habitat use, and individual dietary specialization across time. This approach can enhance our understanding of caribou responses to changing snow regimes, plant phenology, and migratory cues—all of which are critical for adaptive conservation management. Furthermore, broadening the isotopic toolkit (e.g., incorporating δ^18^O, δ^2^H, δ^34^S, and δ^44^Ca) will strengthen dietary reconstruction and refine our understanding of how physiological and environmental variables shape antler chemistry. Ultimately, further isotope studies of antler will be integral for monitoring caribou ecological shifts and framing proactive strategies for sustaining caribou populations in a rapidly changing Arctic landscape.

## Author Contributions

**Matthew Brenning:** conceptualization (equal), formal analysis (lead), investigation (equal), methodology (equal), software (lead), writing – original draft (lead), writing – review and editing (equal). **Fred J. Longstaffe:** data curation (equal), funding acquisition (equal), methodology (equal), resources (equal), writing – original draft (supporting), writing – review and editing (supporting). **Joshua H. Miller:** formal analysis (supporting), investigation (supporting), writing – review and editing (equal). **Danielle Fraser:** conceptualization (equal), formal analysis (equal), funding acquisition (equal), methodology (equal), supervision (equal), writing – original draft (equal), writing – review and editing (supporting).

## Conflicts of Interest

The authors declare no conflicts of interest.

## Supporting information


**Data S1:** ece372294‐sup‐0001‐DataS1.R.

**Appendix S1:** ece372294‐sup‐0002‐AppendixS1.docx.

## Data Availability

The datasets used and RStudio code used in this study are available with the corresponding Supporting Information.
