# Supplementary material for: Stable Isotopes of Carbon and Nitrogen in Female Qamanirjuaq Caribou (Rangifer tarandus groenlandicus) Antlers in Relation to Diet and Physiology
Source: Ecol Evol. 2025 Oct 7;15(10):e72294. doi: 10.1002/ece3.72294 (PMC12502633; doi:10.1002/ece3.72294)
Supplement: Supplementary file 2 — Appendix S1: ece372294‐sup‐0002‐AppendixS1.docx. [file ECE3-15-e72294-s001.docx]

Stable isotope of carbon and nitrogen in female Qamanirjuaq caribou (*Rangifer tarandus groenlandicus*) antlers in relation to diet and physiology

Matthew Brenning^1,2*^, Fred J Longstaffe^3^, Joshua H. Miller^5,6*,^ Danielle Fraser^1,2,4,5^

^1^Department of Earth Sciences, Carleton University, Ottawa, Ontario, Canada.

^2^Palaeobiology, Canadian Museum of Nature, Ottawa, Ontario, Canada.

^3^Department of Earth Sciences, The University of Western Ontario, Ontario, Canada.

^4^Department of Biology, Carleton University, Ottawa, Ontario, Canada.

^5^Department of Paleobiology, Smithsonian National Museum of Natural History, Washington, DC, United States.

^6^Department of Geosciences, University of Cincinnati, Cincinnati, Ohio, United States.

*Corresponding author

E-mail: matthewbrenning@cmail.carleton.ca (MB)

Author Contributions: MB and DR conceived and designed the study. MB performed the sample preparation. FJL conducted the isotope analysis. MB, FJL, and DF wrote the manuscript. JM assisted in interpreting results and edited the manuscript.

**Supplementary Table 1.** Data from the Canadian Wildlife Services study for the 19 female caribou selected for study

| **CMN Catalogue Number** | **Date Collected** | **Latitude** | **Longitude** | **Weight (kg)** | **Tot Len (cm)** | **Tail (cm)** | **Hind Foot (cm)** | **Ear (cm)** | **Reproductive Condition** |
| --- | --- | --- | --- | --- | --- | --- | --- | --- | --- |
| CMN 39077 | 17-Sep-67 | 61.15 | –95.4167 | 95 | 165 | 10 | 50 | 12 | Lactating |
| CMN 39080 | 17-Sep-67 | 61.15 | –95.4167 | 70 | 154 | 12 | 51 | 12 | Dry |
| CMN 39084 | 18-Sep-67 | 61.25 | –95.4167 | 94 | 169 | 16 | 50.5 | 11 | Lactating |
| CMN 39085 | 18-Sep-67 | 61.25 | –95.4167 | 82 | 147 | 11.5 | 50 | 11 | Dry |
| CMN 39086 | 18-Sep-67 | 61.25 | –95.4167 | 86 | 166 | 14 | 54 | 11.5 | Dry |
| CMN 39091 | 18-Sep-67 | 61.25 | –95.35 | 86 | 156 | 12 | - | 12 | Dry |
| CMN 39092 | 18-Sep-67 | 61.25 | –95.35 | 85 | 165 | 15.5 | 52 | 11 | Lactating |
| CMN 39096 | 18-Sep-67 | 61.25 | –95.35 | 85 | 163 | 12 | 50 | 11 | Dry |
| CMN 39097 | 18-Sep-67 | 61.25 | –95.35 | 99 | 166.5 | 11 | 51 | 12 | Lactating |
| CMN 39101 | 18-Sep-67 | 61.25 | –95.35 | 67 | 148 | 11 | 51 | 11.5 | Dry |
| CMN 39103 | 18-Sep-67 | 61.25 | –95.35 | 89 | 165 | 11 | 51.5 | 11.5 | Dry |
| CMN 39112 | 19-Sep-67 | 61.2 | –95.25 | 93 | 172 | 13.5 | 50.5 | 12 | Dry |
| CMN 39113 | 19-Sep-67 | 61.2 | –95.25 | 84 | 160 | 11 | 48 | 11.5 | Dry |
| CMN 39114 | 19-Sep-67 | 61.25 | –95.3167 | 96 | 163 | 12 | 52 | 12 | Lactating |
| CMN 39117 | 19-Sep-67 | 61.25 | –95.3167 | 87 | 164 | 11 | 51.5 | 12.5 | Lactating |
| CMN 39125 | 19-Sep-67 | 61.25 | –95.3167 | 86 | 157.5 | 14 | 49 | 11.5 | Lactating |
| CMN 39130 | 19-Sep-67 | 61.25 | –95.3167 | 87.5 | 167.5 | 13 | 51 | 11 | Dry |
| CMN 39133 | 19-Sep-67 | 61.25 | –95.3167 | 85 | 162 | 14 | 49 | 12 | Lactating |
| CMN 39140 | 21-Sep-67 | 61.2 | –95.3167 | 80 | 166 | 15 | 51 | 12 | Dry |

**Supplementary Table 2.** Stable carbon and nitrogen isotope and elemental data for collagen obtained from sample female antlers. The number and suffix A representing the sample location along the antler beam.

|  | | ***δ*^13^C_col_** |  | ***δ*^15^N_col_** |  |  |
| --- | --- | --- | --- | --- | --- | --- |
| **Num** | **Sample** | **(VPDB, ‰)** | **C (wt%)** | **(AIR, ‰)** | **N (wt%)** | **C:N** |
| 1 | 39077-1A | –18.83 | 33.84 | +3.72 | 11.70 | 3.37 |
| 2 | 39077-2A | –18.78 | 31.90 | +3.71 | 11.06 | 3.36 |
| 3 | 39077-3A | –18.67 | 38.74 | +4.13 | 13.59 | 3.33 |
| 4 | 39077-4A | –18.69 | 34.59 | +4.12 | 11.98 | 3.37 |
| 5 | 39077-5A | –18.67 | 33.03 | +4.28 | 11.42 | 3.37 |
| 6 | 39077-6A | –18.63 | 45.49 | +4.18 | 16.13 | 3.29 |
| 7 | 39077-7A | –18.64 | 41.35 | +4.11 | 14.36 | 3.36 |
| 8 | 39077-8A | –18.49 | 44.90 | +3.88 | 15.53 | 3.37 |
| 9 | 39077-9A | –19.03 | 46.40 | +4.05 | 15.23 | 3.55 |
| 10 | 39080-1A | –19.02 | 44.24 | +4.85 | 15.53 | 3.32 |
| 11 | 39080-2A | –18.93 | 41.53 | +5.53 | 14.81 | 3.27 |
| 12 | 39080-3A | –18.86 | 41.45 | +6.00 | 14.72 | 3.28 |
| 13 | 39080-4A | –18.75 | 29.42 | +5.75 | 10.35 | 3.31 |
| 14 | 39080-5A | –18.70 | 41.99 | +5.59 | 14.55 | 3.36 |
| 15 | 39080-6A | –18.58 | 58.01 | +5.44 | 19.72 | 3.43 |
| 16 | 39081-1A | –18.85 | 41.43 | +4.43 | 14.56 | 3.32 |
| 17 | 39081-2A | –18.85 | 40.83 | +4.86 | 14.52 | 3.28 |
| 18 | 39081-3A | –18.92 | 41.82 | +4.44 | 14.97 | 3.26 |
| 19 | 39081-4A | –18.95 | 41.66 | +5.16 | 14.59 | 3.33 |
| 20 | 39081-5A | –18.67 | 41.90 | +5.67 | 14.92 | 3.28 |
| 21 | 39081-5A dup | –18.65 | 41.13 | +5.62 | 14.53 | 3.30 |
| 22 | 39081-6A | –18.55 | 42.03 | +5.75 | 14.80 | 3.31 |
| 23 | 39081-7A | –18.31 | 43.71 | +5.62 | 15.07 | 3.38 |
| 24 | 39084-1A | –19.43 | 40.30 | +2.25 | 14.19 | 3.31 |
| 25 | 39084-2A | –19.22 | 40.33 | +2.31 | 14.46 | 3.25 |
| 26 | 39084-3A | –19.31 | 40.59 | +2.78 | 14.53 | 3.26 |
| 27 | 39084-4A | –19.15 | 40.97 | +2.97 | 14.55 | 3.28 |
| 28 | 39084-5A | –19.07 | 40.98 | +3.29 | 14.58 | 3.28 |
| 29 | 39084-6A | –18.94 | 41.75 | +3.27 | 14.68 | 3.32 |
| 30 | 39084-6A dup | –18.91 | 42.27 | +3.39 | 14.97 | 3.29 |
| 31 | 39084-7A | –18.72 | 42.15 | +3.25 | 14.70 | 3.34 |
| 32 | 39084-8A | –18.44 | 42.15 | +3.33 | 14.58 | 3.37 |
| 33 | 39085-1A | –19.94 | 42.03 | +4.17 | 14.42 | 3.40 |
| 34 | 39085-2A | –19.49 | 45.49 | +4.33 | 15.95 | 3.33 |
| 35 | 39085-3A | –19.42 | 41.65 | +4.27 | 14.85 | 3.27 |
| 36 | 39085-4A | –19.43 | 42.16 | +4.50 | 14.69 | 3.35 |
| 37 | 39085-5A | –19.50 | 42.67 | +4.64 | 14.68 | 3.39 |
| 38 | 39085-6A | –19.40 | 43.15 | +4.23 | 14.61 | 3.44 |
| 39 | 39086-1A | –19.30 | 42.85 | +3.20 | 15.04 | 3.32 |
| 40 | 39086-2A | –19.11 | 41.85 | +3.70 | 14.95 | 3.26 |
| 41 | 39086-3A | –19.05 | 41.72 | +3.85 | 14.84 | 3.28 |
| 42 | 39086-3A dup | –19.08 | 42.00 | +3.86 | 14.95 | 3.28 |
| 43 | 39086-4A | –18.87 | 41.69 | +4.00 | 14.88 | 3.27 |
| 44 | 39086-5A | –18.96 | 42.53 | +3.92 | 14.97 | 3.31 |
| 45 | 39086-6A | –18.93 | 42.07 | +3.69 | 14.59 | 3.36 |
| 46 | 39086-7A | –18.83 | 42.87 | +3.66 | 14.57 | 3.43 |
| 47 | 39091-1A | –19.72 | 47.57 | +3.98 | 16.42 | 3.38 |
| 48 | 39091-1A dup | –19.76 | 45.08 | +3.98 | 15.54 | 3.38 |
| 49 | 39091-2A | –19.28 | 44.85 | +4.33 | 15.97 | 3.27 |
| 50 | 39091-3A | –19.06 | 54.62 | +5.10 | 19.47 | 3.27 |
| 51 | 39091-4A | –18.99 | 44.29 | +5.49 | 15.72 | 3.29 |
| 52 | 39091-5A | –19.37 | 33.26 | +5.00 | 11.43 | 3.39 |
| 53 | 39091-6A | –19.55 | 43.78 | +4.68 | 15.41 | 3.31 |
| 54 | 39091-7A | –19.41 | 44.91 | +4.89 | 15.74 | 3.33 |
| 55 | 39091-8A | –19.15 | 39.06 | +5.99 | 13.27 | 3.43 |
| 56 | 39092-1A | –19.49 | 44.95 | +6.07 | 14.72 | 3.56 |
| 57 | 39092-2A | –18.76 | 41.98 | +6.69 | 14.67 | 3.34 |
| 58 | 39092-3A | –18.61 | 42.34 | +6.64 | 14.91 | 3.31 |
| 59 | 39092-4A | –18.80 | 42.30 | +6.63 | 14.75 | 3.35 |
| 60 | 39092-5A | –18.96 | 42.54 | +6.73 | 14.69 | 3.38 |
| 61 | 39092-6A | –19.05 | 43.01 | +6.86 | 14.68 | 3.42 |
| 62 | 39092-7A | –18.89 | 43.00 | +6.99 | 14.57 | 3.44 |
| 63 | 39092-8A | –19.72 | 41.33 | +6.57 | 13.13 | 3.67 |
| 64 | 39096-1A | –18.91 | 42.15 | +4.09 | 15.12 | 3.25 |
| 65 | 39096-1A dup | –18.96 | 42.14 | +3.87 | 15.17 | 3.24 |
| 66 | 39096-2A | –18.82 | 44.98 | +4.37 | 15.98 | 3.28 |
| 67 | 39096-3A | –18.46 | 42.63 | +5.61 | 15.18 | 3.27 |
| 68 | 39096-4A | –18.40 | 44.98 | +5.42 | 15.89 | 3.30 |
| 69 | 39096-5A | –18.71 | 42.41 | +5.34 | 15.14 | 3.27 |
| 70 | 39096-6A | –18.81 | 42.50 | +5.56 | 14.93 | 3.32 |
| 71 | 39096-7A | –18.57 | 42.48 | +5.86 | 15.00 | 3.30 |
| 72 | 39097-1A | –19.25 | 42.79 | +5.52 | 14.93 | 3.34 |
| 73 | 39097-2A | –19.15 | 46.70 | +5.59 | 15.97 | 3.41 |
| 74 | 39097-3A | –18.78 | 41.83 | +5.82 | 14.64 | 3.33 |
| 75 | 39097-4A | –18.73 | 42.66 | +5.75 | 14.83 | 3.35 |
| 76 | 39097-5A | –18.80 | 42.02 | +5.64 | 14.52 | 3.40 |
| 77 | 39097-5A dup | –21.79 | 42.05 | +5.71 | 14.50 | 3.37 |
| 78 | 39097-5A dup | –18.89 | 42.97 | +5.44 | 14.73 | 3.40 |
| 79 | 39097-6A | –18.68 | 41.59 | +5.57 | 14.56 | 3.33 |
| 80 | 39097-7A | –22.37 | 42.67 | +5.59 | 14.48 | 3.33 |
| 81 | 39097-7A dup | –19.25 | 43.27 | +5.68 | 14.67 | 3.44 |
| 82 | 39097-8A | –19.16 | 46.33 | +5.47 | 15.08 | 3.58 |
| 83 | 39097-8A dup | –19.31 | 46.92 | +5.48 | 15.44 | 3.54 |
| 84 | 39101-1A | –19.19 | 41.35 | +5.57 | 14.55 | 3.31 |
| 85 | 39101-2A | –18.83 | 41.43 | +6.14 | 14.88 | 3.25 |
| 86 | 39101-3A | –18.63 | 41.71 | +6.93 | 14.81 | 3.28 |
| 87 | 39101-4A | –18.49 | 42.15 | +6.97 | 14.74 | 3.33 |
| 88 | 39101-5A | –18.90 | 35.29 | +7.27 | 11.95 | 3.44 |
| 89 | 39101-6A | –18.36 | 42.02 | +5.91 | 14.45 | 3.39 |
| 90 | 39103-1A | –19.32 | 45.38 | +6.05 | 15.95 | 3.32 |
| 91 | 39103-2A | –19.18 | 44.97 | +6.40 | 15.69 | 3.34 |
| 92 | 39103-3A | –19.01 | 44.65 | +7.03 | 15.14 | 3.44 |
| 93 | 39103-4A | –18.82 | 41.25 | +6.96 | 14.17 | 3.40 |
| 94 | 39103-4A dup | –18.80 | 41.30 | +6.89 | 14.28 | 3.37 |
| 95 | 39103-5A | –18.82 | 44.57 | +6.86 | 15.49 | 3.35 |
| 96 | 39103-6A | –19.47 | 45.39 | +6.51 | 15.26 | 3.47 |
| 97 | 39103-7A | –19.69 | 45.02 | +6.74 | 14.51 | 3.62 |
| 98 | 39112-1A | –19.70 | 42.76 | +3.44 | 14.78 | 3.37 |
| 99 | 39112-2A | –19.36 | 41.72 | +3.56 | 14.74 | 3.30 |
| 100 | 39112-3A | –19.31 | 41.80 | +3.41 | 14.88 | 3.28 |
| 101 | 39112-4A | –19.32 | 41.85 | +3.61 | 14.72 | 3.32 |
| 102 | 39112-4A dup | –19.26 | 41.85 | +3.68 | 14.71 | 3.32 |
| 103 | 39112-5A | –19.47 | 42.34 | +3.88 | 14.61 | 3.38 |
| 104 | 39112-6A | –18.95 | 42.37 | +4.03 | 14.90 | 3.32 |
| 105 | 39112-7A | –18.63 | 42.11 | +4.17 | 14.62 | 3.36 |
| 106 | 39113-1A | –18.97 | 43.80 | +5.22 | 15.29 | 3.34 |
| 107 | 39113-2A | –18.82 | 44.78 | +5.47 | 15.51 | 3.37 |
| 108 | 39113-3A | –18.79 | 40.12 | +5.42 | 13.81 | 3.39 |
| 109 | 39113-4A | –18.97 | 44.04 | +5.89 | 14.97 | 3.43 |
| 110 | 39113-5A | –18.62 | 38.90 | +5.77 | 13.36 | 3.40 |
| 111 | 39113-6A | –18.64 | 40.33 | +5.88 | 13.85 | 3.40 |
| 112 | 39113-7A | –18.84 | 37.24 | +6.56 | 12.31 | 3.53 |
| 113 | 39113-7A dup | –18.84 | 46.67 | +6.62 | 15.48 | 3.52 |
| 114 | 39114-1A | –19.37 | 40.89 | +4.32 | 14.05 | 3.39 |
| 115 | 39114-2A | –18.72 | 43.32 | +4.47 | 15.44 | 3.27 |
| 116 | 39114-3A | –18.71 | 36.61 | +4.46 | 12.65 | 3.37 |
| 117 | 39114-4A | –18.59 | 43.57 | +4.75 | 15.35 | 3.31 |
| 118 | 39114-5A | –18.65 | 44.60 | +4.74 | 15.47 | 3.36 |
| 119 | 39114-6A | –18.55 | 44.76 | +4.65 | 15.58 | 3.35 |
| 120 | 39114-8A | –18.30 | 44.70 | +5.14 | 15.21 | 3.43 |
| 121 | 39117-1A | –19.18 | 41.43 | +4.21 | 14.87 | 3.25 |
| 122 | 39117-2A | –18.92 | 41.58 | +4.40 | 14.96 | 3.24 |
| 123 | 39117-3A | –18.81 | 41.50 | +4.52 | 14.91 | 3.25 |
| 124 | 39117-4A | –18.75 | 41.94 | +4.82 | 14.99 | 3.26 |
| 125 | 39117-5A | –18.44 | 41.92 | +5.19 | 14.93 | 3.28 |
| 126 | 39117-6A | –18.52 | 42.09 | +5.27 | 14.90 | 3.29 |
| 127 | 39117-7A | –18.11 | 42.16 | +5.30 | 15.00 | 3.28 |
| 128 | 39125-1A | –19.93 | 42.15 | +4.54 | 13.80 | 3.56 |
| 129 | 39125-2A | –19.34 | 41.64 | +4.69 | 14.86 | 3.27 |
| 130 | 39125-3A | –19.36 | 33.91 | +5.01 | 11.39 | 3.47 |
| 131 | 39125-4A | –19.10 | 41.29 | +5.15 | 14.88 | 3.24 |
| 132 | 39125-5A | –19.31 | 41.43 | +6.03 | 14.58 | 3.31 |
| 133 | 39125-6A | –19.20 | 45.25 | +4.87 | 15.52 | 3.40 |
| 134 | 39125-7A | –18.95 | 39.76 | +4.99 | 13.95 | 3.32 |
| 135 | 39130-1A | –18.42 | 44.54 | +5.66 | 15.46 | 3.36 |
| 136 | 39130-2A | –18.68 | 47.01 | +5.21 | 16.74 | 3.27 |
| 137 | 39130-3A | –18.14 | 45.20 | +5.55 | 15.87 | 3.32 |
| 138 | 39130-4A | –18.16 | 45.61 | +5.59 | 16.15 | 3.29 |
| 139 | 39130-5A | –18.12 | 45.18 | +5.66 | 16.02 | 3.29 |
| 140 | 39130-6A | –18.39 | 44.88 | +5.59 | 15.69 | 3.34 |
| 141 | 39130-7A | –18.26 | 45.58 | +5.50 | 15.79 | 3.37 |
| 142 | 39130-7A dup | –18.29 | 46.12 | +5.64 | 16.03 | 3.36 |
| 143 | 39130-8A | –18.39 | 41.80 | +5.50 | 13.85 | 3.52 |
| 144 | 39133-1A | –19.44 | 41.90 | +4.77 | 14.78 | 3.34 |
| 145 | 39133-1A dup | –19.57 | 41.66 | +4.62 | 14.57 | 3.31 |
| 146 | 39133-2A | –19.23 | 41.94 | +5.20 | 14.66 | 3.34 |
| 147 | 39133-3A | –19.03 | 41.46 | +5.07 | 14.50 | 3.33 |
| 148 | 39133-4A | –19.14 | 42.11 | +5.16 | 14.69 | 3.34 |
| 149 | 39133-5A | –19.21 | 41.77 | +5.19 | 14.71 | 3.31 |
| 150 | 39133-6A | –19.15 | 42.30 | +5.57 | 14.80 | 3.33 |
| 151 | 39133-7A | –19.25 | 42.30 | +5.96 | 14.64 | 3.37 |
| 152 | 39133-8A | –19.22 | 42.05 | +6.82 | 14.36 | 3.42 |
| 153 | 39133-9A | –18.77 | 42.18 | +5.22 | 14.69 | 3.35 |
| 154 | 39133-9A dup | –18.83 | 48.52 | +5.35 | 17.12 | 3.30 |
| 155 | 39140-1A | –19.52 | 41.54 | +4.93 | 14.75 | 3.28 |
| 156 | 39140-2A | –19.46 | 44.88 | +5.18 | 15.56 | 3.36 |
| 157 | 39140-3A | –19.30 | 41.64 | +5.49 | 14.61 | 3.32 |
| 158 | 39140-3A dup | –19.26 | 41.78 | +5.57 | 14.57 | 3.34 |
| 159 | 39140-4A | –19.20 | 38.86 | +5.64 | 13.81 | 3.28 |
| 160 | 39140-5A | –19.25 | 41.75 | +6.33 | 14.31 | 3.40 |
| 161 | 39140-6A | –18.94 | 41.46 | +5.74 | 14.64 | 3.30 |

**Supplementary Table 3.** Stable carbon (*δ*^13^C) and nitrogen (*δ*^15^N) isotope data for source groups (horsetail, lichen, liverwort, and woody plants), location of collection by the National Herbarium of Canada and fungi data, as reported by Hobbie *et al.* (2017).

|  | | ***δ*^13^C** | ***δ*^15^N** |  |  |  |
| --- | --- | --- | --- | --- | --- | --- |
| **Species** | **CMN#** | **(VPDB, ‰)** | **(AIR, ‰)** | **Group** | **Lat** | **Long** |
| *Equisetum arvense* | CAN 10004683 | –24.07 | +3.64 | Horsetail | 63.83342 | -104.084 |
| *Equisetum scirpoides* | CANL 10004537 | –27.8 | +1.8 | Horsetail | 64.15 | -102.533 |
| *Equisetum variegatum* | CANL 10001236 | –25.6 | +1.0 | Horsetail | 64.15 | -103.533 |
| *Equisetum variegatum* | CAN 10001224 | –25.36 | +10.88 | Horsetail | 64.31667 | -96.05 |
| *Equisetum variegatum* | CAN 10001221 | –26.73 | +9.54 | Horsetail | 64.31667 | -96.05 |
| *Equisetum arvense* | CAN 10004684 | –24.81 | +2.33 | Horsetail | 63.65 | -104.5 |
| *Equisetum arvense* | CAN 10004749 | –23.74 | +3.81 | Horsetail | 63.83333 | -104.083 |
| *Equisetum sylvaticum* | CAN 10004832 | –23.57 | +2.99 | Horsetail | 63.83333 | -104.083 |
| *Equisetum sylvaticum* | CAN 10004827 | –23.36 | +5.03 | Horsetail | 63.83333 | -104.083 |
| *Cladonia amaurocraea* | CANL 89317 | –23.7 | –2.9 | Lichen | 59.45 | -97.7167 |
| *Cladonia amaurocraea* | CANL 89230 | –24.39 | +1.81 | Lichen | 62.21667 | -94.9833 |
| *Cladonia pocillum* | CANL 89231 | –25.04 | –0.71 | Lichen | 62.21667 | -94.9833 |
| *Cladonia rangiferina* | CANL 31444 | –24.64 | –0.70 | Lichen | 64.21667 | -102.117 |
| *Cladonia subfurcata* | CANL 89232 | –22.49 | –0.81 | Lichen | 62.21667 | -94.9833 |
| *Cladonia amaurocraea* | CANL 46908 | –24.1 | +0.5 | Lichen | 63.58333 | -110.033 |
| *Cladonia amaurocraea* | CANL 46908 QCD | –23.8 | –1.8 | Lichen | 63.58333 | -110.033 |
| *Cladonia amaurocraea* | CANL 46938 | –27.27 | –2.46 | Lichen | 60.78333 | -107.633 |
| *Cladonia arbuscula* | CANL 89268 | –25.08 | –0.27 | Lichen | 59.08333 | -102.083 |
| *Cladonia coccifera* | CANL 46910 | –24.82 | 0.32 | Lichen | 63.58333 | -110.033 |
| *Cladonia rangiferina* | CANL 31427 | –24.1 | –0.7 | Lichen | 63.7 | -104.417 |
| *Cladonia stellaris* | CANL 46907 | –23.57 | –0.44 | Lichen | 63.58333 | -110.033 |
| *Cladonia amaurocraea* | CANL 46952 | –23.9 | –0.8 | Lichen | 61.51667 | -108.1 |
| *Cladonia amaurocraea* | CANL 46923 | –24.93 | –1.25 | Lichen | 62.58333 | -109.633 |
| *Cladonia amaurocraea* | CANL 46890 | –24.42 | –0.82 | Lichen | 62.53333 | -108.3 |
| *Cladonia cenotea* | CANL 89249 | –27.59 | –1.44 | Lichen | 59.08333 | -102.083 |
| *Cladonia chlorophaea* | CANL 89472 | –26.92 | +1.34 | Lichen | 59.08333 | -102.083 |
| *Cladonia coccifera* | CANL 46953 | –24.39 | +0.15 | Lichen | 61.51667 | -108.1 |
| *Cladonia coccifera* | CANL 89318 | –24.80 | +1.63 | Lichen | 59.45 | -97.7167 |
| *Cladonia coccifera* | CANL 46892 | –24.48 | +0.54 | Lichen | 60.28333 | -109.033 |
| *Cladonia coccifera* | CANL 89238 | –24.51 | +0.52 | Lichen | 60.08333 | -103.333 |
| *Cladonia cornuta* | CANL 46954 | –25.39 | +1.51 | Lichen | 61.51667 | -108.1 |
| *Cladonia crispata* | CANL 46933 | –24.27 |  | Lichen | 60.78333 | -107.633 |
| *Cladonia crispata* | CANL 89808 | –24.30 | –0.46 | Lichen | 59.75417 | -103.333 |
| *Cladonia cristatella* | CANL 89250 | –22.93 | –1.84 | Lichen | 59.08333 | -102.083 |
| *Cladonia deformis* | CANL 46955 | –25.01 | +1.61 | Lichen | 61.51667 | -108.1 |
| *Cladonia deformis* | CANL 46919 | –26.11 | –1.29 | Lichen | 62.58333 | -109.633 |
| *Cladonia gracilis* | CANL 46891 | –23.85 | –2.81 | Lichen | 60.28333 | -109.033 |
| *Cladonia gracilis* | CANL 46927 | –21.31 | –2.76 | Lichen | 62.58333 | -109.633 |
| *Cladonia gracilis* | CANL 46934 | –25.08 | –3.92 | Lichen | 60.78333 | -107.633 |
| *Cladonia macrophylla* | CANL 89297 | –25.17 | +1.89 | Lichen | 59.55 | -97.8 |
| *Cladonia macrophylla* | CANL 89239 | –25.00 | +0.90 | Lichen | 60.08333 | -103.333 |
| *Cladonia phyllophora* | CANL 89807 | –24.05 | +2.43 | Lichen | 59.75417 | -103.333 |
| *Cladonia phyllophora* | CANL 89474 | –22.58 | +1.56 | Lichen | 59.45 | -97.7333 |
| *Cladonia rangiferina* | CANL 46928 | –23.9 | +0.4 | Lichen | 61.51667 | -108.1 |
| *Cladonia rangiferina* | CANL 46940 | –25.03 | –3.62 | Lichen | 60.78333 | -107.633 |
| *Cladonia rangiferina* | CANL 46925 | –24.1 | –6.0 | Lichen | 62.58333 | -109.633 |
| *Cladonia rangiferina* | CANL 89245 | –24.64 | +1.06 | Lichen | 59.08333 | -102.083 |
| *Cladonia rangiferina* | CANL 46924 | –25.13 | –1.32 | Lichen | 62.58333 | -109.633 |
| *Cladonia stellaris* | CANL 89247 | –24.1 | –4.1 | Lichen | 59.08333 | -102.083 |
| *Cladonia stellaris* | CANL 46956 | –24.8 | –3.7 | Lichen | 61.51667 | -108.1 |
| *Cladonia stellaris* | CANL 46926 | –26.31 | –0.95 | Lichen | 62.58333 | -109.633 |
| *Cladonia stellaris* | CANL 46939 | –24.76 | –1.46 | Lichen | 60.78333 | -107.633 |
| *Cladonia subfurcata* | CANL 89319 | –23.3 | –3.5 | Lichen | 59.45 | -97.7167 |
| *Cladonia subfurcata* | CANL 89319 QCD | –23.3 | –3.2 | Lichen | 59.45 | -97.7167 |
| *Cladonia symphycarpa* | CANL 89809 | –25.41 | +1.12 | Lichen | 59.75417 | -103.333 |
| *Ptilidium ciliare* | CANL 13492 | –27.6 | –5.0 | Liverwort | 62.58333 | -109.633 |
| *Ptilidium ciliare* | CANL 21717 | –29.6 | –1.5 | Liverwort | 59.08333 | -102.083 |
| *Ptilidium ciliare* | CANL 25511 | –28.8 | +0.2 | Liverwort | 60.91667 | -110.5 |
| *Ptilidium ciliare* | CANL 13491 | –28.2 | –4.6 | Liverwort | 62.58333 | -109.633 |
| *Larix laricina* | CAN 10005691 | –25.58 | –1.95 | Woody | 64.13333 | -102.533 |
| *Larix laricina* | CANL 10005686 | –28.2 | –3.0 | Woody | 64.13333 | -102.533 |
| *Picea glauca* | CAN 10005822 | –26.12 | –7.79 | Woody | 64.21667 | -102.117 |
| *Picea glauca* | CANL 10005826 | –26.2 | –3.0 | Woody | 63.63333 | -104.65 |
| *Picea glauca* | CAN 10005807 | –27.43 | –3.57 | Woody | 63.95 | -103.883 |
| *Picea mariana* | CANL 10006171 | –26.5 | –6.1 | Woody | 63.63333 | -104.65 |
| *Picea mariana* | CAN 10006162 | –26.74 | –6.17 | Woody | 63.61667 | -104.517 |
| *Picea mariana* | CANL 10006163 | –28.3 | –7.9 | Woody | 63.63333 | -104.65 |
| *Rhododendron groenlandicum* | CAN 10076271 | –25.61 | –1.33 | Woody | 64.13333 | -102.533 |
| *Rhododendron tomentosum* | CANL 10076538 | –27.6 | –5.4 | Woody | 64.21667 | -102.117 |
| *Vaccinium uliginosum* | CAN 10078701 | –26.04 | –1.56 | Woody | 64.18333 | -103.683 |
| *Rhododendron groenlandicum* | CANL 10076270 | –28.6 | –2.4 | Woody | 63.95 | -103.883 |
| *Rhododendron groenlandicum* | CANL 10076268 | –27.2 | –7.6 | Woody | 63.63333 | -104.683 |
| *Rhododendron tomentosum* | CANL 10076539 | –27.9 | –3.0 | Woody | 63.95 | -103.883 |
| *Rhododendron tomentosum* | CANL 10076531 | –27.3 | +1.4 | Woody | 63.61667 | -104.517 |
| *Rhododendron tomentosum* | CANL 10075017 | –27.7 | –3.2 | Woody | 63.63333 | -104.65 |
| *Rhododendron tomentosum* | CAN 10076543 | –26.27 | –4.48 | Woody | 63.63333 | -104.683 |
| *Betula glandulosa* | CAN 10026784 | –24.89 | +0.21 | Woody | 63.65 | -104.5 |
| *Betula glandulosa* | CAN 10026782 | –25.24 | –1.71 | Woody | 63.95 | -103.883 |
| *Betula glandulosa* | CAN 10026785 | –26.12 | –0.81 | Woody | 63.95 | -103.883 |
| *Betula glandulosa* | CAN 10026717 | –24.87 | –0.06 | Woody | 63.65 | -104.5 |
| **Hobbie et al. (2017)** | | | | | | |
| **Taxon** | **Location** |  |  |  |  |  |
| *Boletaceae* | Atigun | –25.3 | +8.7 | Fungi |  |  |
| *Boletus* | Atigun | –24.9 | +9.8 | Fungi |  |  |
| *Leccinum* | Atigun | –25.0 | +6.6 | Fungi |  |  |
| *Laccaria* | Atigun | –25.2 | +2.8 | Fungi |  |  |
| *Lactarius* | Atigun | –25.7 | +4.9 | Fungi |  |  |
| *Russula* | Atigun | –25.6 | +5.8 | Fungi |  |  |
| *Boletaceae* | Toolik Lake | –25.5 | +6.3 | Fungi |  |  |
| *Bpletus* | Toolik Lake | –22.7 | +2.0 | Fungi |  |  |
| *Leccinum* | Toolik Lake | –25.4 | +6.5 | Fungi |  |  |
| *Laccaria* | Toolik Lake | –25.3 | +1.3 | Fungi |  |  |
| *Lactarius* | Toolik Lake | –25.4 | +3.0 | Fungi |  |  |
| *Russula* | Toolik Lake | –26.8 | +4.9 | Fungi |  |  |

**Supplementary Table 4.** Linear regressions for *δ*^13^C_col_ and *δ*^15^N_col_ variation along antler length for all caribou separated by reproductive status.

| **Reproductive Status** | **Isotope** |  | **Coefficients** | **Standard Error** | **T Stat** | **P-value** | **Residuals** | | |
| --- | --- | --- | --- | --- | --- | --- | --- | --- | --- |
|  |  |  |  |  |  |  | **Min** | **Median** | **Max** |
| All | *δ*^13^C_col_ | Intercept | –19.00405 | 0.02791 | –680.928 | < 0.0001 | –0.93595 | 0.01925 | 0.80823 |
| Caribou |  | X Variable | 0.19250 | 0.04744 | 4.058 | < 0.0001 |  |  |  |
|  | *δ*^15^N_col_ | Intercept | 4.5677 | 0.0800 | 57.099 | < 0.0001 | –2.5868 | 0.1106 | 2.1444 |
|  |  | X Variable | 0.09634 | 0.01360 | 7.085 | < 0.0001 |  |  |  |
| Males | *δ*^13^C_col_ | Intercept | –18.92728 | 0.03242 | –583.882 | < 0.0001 | –0.81053 | 0.00789 | 0.60296 |
|  |  | X Variable | 0.09034 | 0.05570 | 1.622 | 0.106 |  |  |  |
|  | *δ*^15^N_col_ | Intercept | 4.5246 | 0.0955 | 47.378 | < 0.0001 | –2.58812 | 0.09939 | 1.94836 |
|  |  | X Variable | 1.0412 | 0.1641 | 6.345 | < 0.0001 |  |  |  |
| Female’s | *δ*^13^C_col_ | Intercept | –19.15200 | 0.07193 | –266.254 | < 0.0001 | –0.93904 | –0.02472 | 0.67096 |
| Lactating |  | X Variable | 0.37105 | 0.11867 | 3.127 | 0.00263 |  |  |  |
|  | *δ*^15^N_col_ | Intercept | 4.5488 | 0.2404 | 18.925 | < 0.0001 | –2.33340 | 0.04878 | 2.04660 |
|  |  | X Variable | 0.6760 | 0.3965 | 1.705 | 0.093 |  |  |  |
| Female’s | *δ*^13^C_col_ | Intercept | –19.14792 | 0.07284 | –262.87 | < 0.0001 | –0.91439 | 0.01177 | 0.89995 |
| Dry |  | X Variable | 0.37231 | 0.12165 | 3.06 | 0.00292 |  |  |  |
|  | *δ*^15^N_col_ | Intercept | 4.7127 | 0.1813 | 25.995 | < 0.0001 | –2.0039 | 0.1373 | 2.0034 |
|  |  | X Variable | 0.9512 | 0.3028 | 3.142 | 0.00228 |  |  |  |


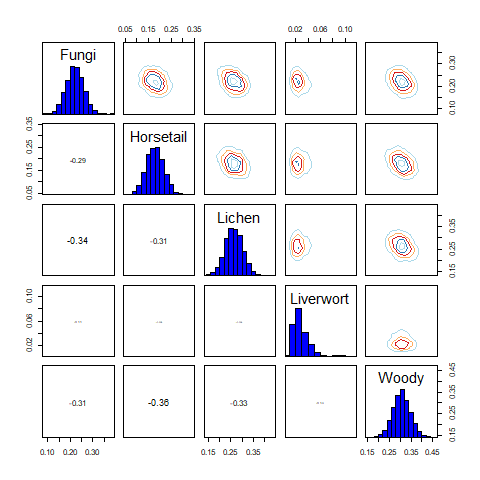


**Supplementary Figure 1A.** Matrix plot for Bayesian stable isotope mixing model using all female antler tissue with uninformative priors and no effects.


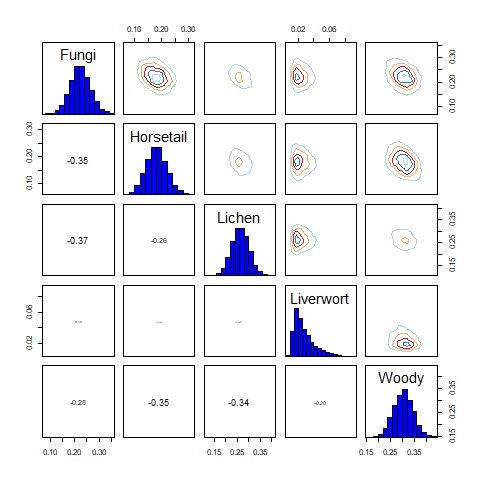


**Supplementary Figure 1B.** Matrix plot for Bayesian stable isotope mixing model using all female antler tissue with uninformative priors and antler length as a continuous covariate.


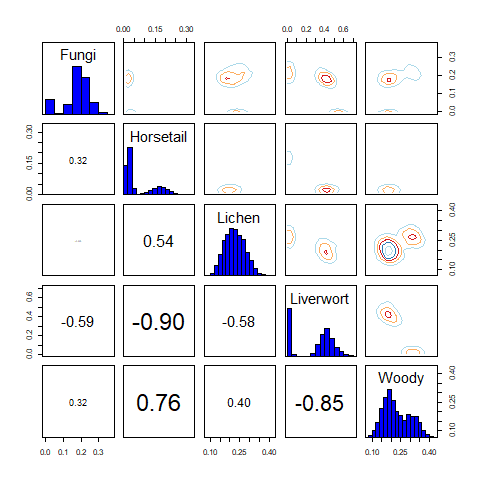


**Supplementary Figure 1C.** Matrix plot for Bayesian stable isotope mixing model using all female antler tissue with informative priors and no effects.


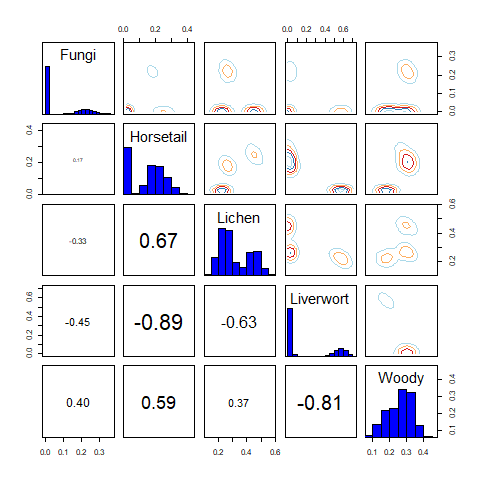


**Supplementary Figure 1D.** Matrix plot for Bayesian stable isotope mixing model using all female antler tissue with informative priors and antler length as a continuous covariate.
